# Epimedin C protects dexamethasone-induced osteoblasts through NRF1/RhoA pathway

**DOI:** 10.18632/aging.204588

**Published:** 2023-03-14

**Authors:** Mi Huang, Lei Yu, Ying Wang, Chunlin Yang

**Affiliations:** 1 Wuhan Hospital of Traditional Chinese and Western Medicine, Wuhan, Hubei 430022, China; 2 College of Life Science Gannan Normal University, Jiangxi 341000, China; 3 Key Laboratory of South China Agricultural Plant Molecular Analysis and Genetic Improvement, Provincial Key Laboratory of Digital Botanical Garden and Public Science, South China Botanical Garden, Chinese Academy of Sciences, Guangzhou 510650, China; 4 Wuhan Red Cross Hospital, Wuhan, Hubei 430000, China; 5 Medical Sciences Building MA202, School of Medicine, University of Missouri, Columbia, MO 65212, USA

**Keywords:** osteoporosis, epimedin C, dexamethasone, RhoA, NRF1

## Abstract

Osteoporosis (OP) is a metabolic bone disease that leads to decrease of bone strength and increase bone brittle and fracture. Dexamethasone (DXMS) usage is a common risk factor of OP. In present study, we found that the Epimedin C protect the DXMS-induced OP, Ras Homolog Family Member A transforming protein (RhoA) was increased in osteoblasts (OBs) and OP models. We further revealed that Nrf1 is a transcription factor that responds to Epimedin C and DXMS in modulating RhoA promoter. The results collectively demonstrate that Epimedin C functions as a positive modifier of RhoA via alteration of Nrf1 transcriptional activity on RhoA promoter, thereby, protecting OBs against OP. Our work is the first study identifying the Epimedin C function in balancing the OBs in OP model via Nrf1-RhoA.

## INTRODUCTION

Osteoporosis (OP) is a metabolic bone disease that occurs when bone resorption outpaces formation during bone remodeling. OP is defined by a progressive decline in bone mass and quality that leads to the decrease of bone strength, and increase of bone brittle and fracture [1–3]. Aging, dexamethasone (DXMS) usage and anticancer treatment are major risk factors for OP [4–6]. DXMS is usually used as an artificial synthetic glucocorticoid for the treatment of psoriasis, allergies, and chronic obstructive pulmonary diseases [7, 8]. This treatment induces cell apoptosis, reactive oxygen species production and endoplasmic reticulum stress, leading to side effects and inducing disease [9, 10]. The pharmacological strategy is to provide adequate vitamin D and calcium and combined with osteogenic drugs to promote the formation of bone structure, or anti-osteoclast drugs to prevent bone density loss [11].

Ras Homolog Family Member A transforming protein (RhoA), a small GTPase that cycles between an active GTP-bound and an inactive GDP-bound state, is mainly associated with cytoskeleton organization [12–14]. RhoA encodes fundamental cellular processes such as cell differentiation, migration, assembly, and organization of the actin cytoskeleton [15, 16], and is vital for bone resorption. RhoA inhibits hypoxia-induced apoptosis and mitochondrial dysfunction in chondrocytes by positively regulating CREB phosphorylation in the osteoblasts (OBs) [17–20].

Nrf1, known as nuclear factor erythroid derived 2-related factor 1 (NFE2L1/TCF11/LCR-F1), encoded by the *nfe2l1* gene, is a cap ‘n’ collar (CNC) bZIP family transcription factor that translocates to the nucleus and regulates target gene expression in response to various stresses [21, 22]. Nrf1 plays a vital role in bone development. Nrf1 is produced by OBs and regulates osterix expression, OB differentiation, and bone formation [23, 24], and also activates the expression of SP7/Osterix in OBs in response to ascorbic acid [25]. Disrupted NFE2L1, specifically in OBs, leads to decreased osterix expression by 57% in the bones of NFE2L1 KO mice [26] showing that Nrf1 is a key regulator of healthy OB function. However, the function of Nrf1 still remains unclear in OP and needs to be explored.

Epimedin C is a major flavonoid in Epimedium species and is the only known chemical marker for the quality control of *E*. wushanense Folium and in some other Herba Epimedii. In previous studies, Epimedin C levels in herbal medicines have been reported to range from 1.22–40.63 mg/g [27], revealing that the Epimedin C extracted from Herba Epimedii can attenuate bone loss associated with estrogen deficiency in mice and humans [28]. It was determined that Epimedin C promotes vascularization during BMP2-induced osteogenesis [29]. Those trials suggested the potential therapeutic applications of Epimedin C and its use as an alternative treatment for OP. However, the specific molecular mechanisms and pathways have rarely been investigated. Thus, further study is needed.

In our study, we used DXMS-induced OBs and a rodent model to evaluate the role of Epimedin C in DXMS-induced OP and identified the related target genes and proteins. Our results showed that the RhoA level was significantly affected by DXMS, but Epimedin C effectively neutralizes the effects of DXMS on RhoA expression and protects OBs from cell death. We further explored the significant role that Epimedin C played in upstream of RhoA by stabilizing healthy OB functions at the RhoA transcriptional level by recovering the transcription activity of Nrf1 on the promoter region of RhoA. The function of Nrf1 in balancing OBs in the OP model was identified and the relationship between Epimedin C and the Nrf1-RhoA pathway in OB was defined for the first time.

## RESULTS

### Epimedin C enhances the migration/proliferation of OB after DXMS-treatment

**Figure 1:**
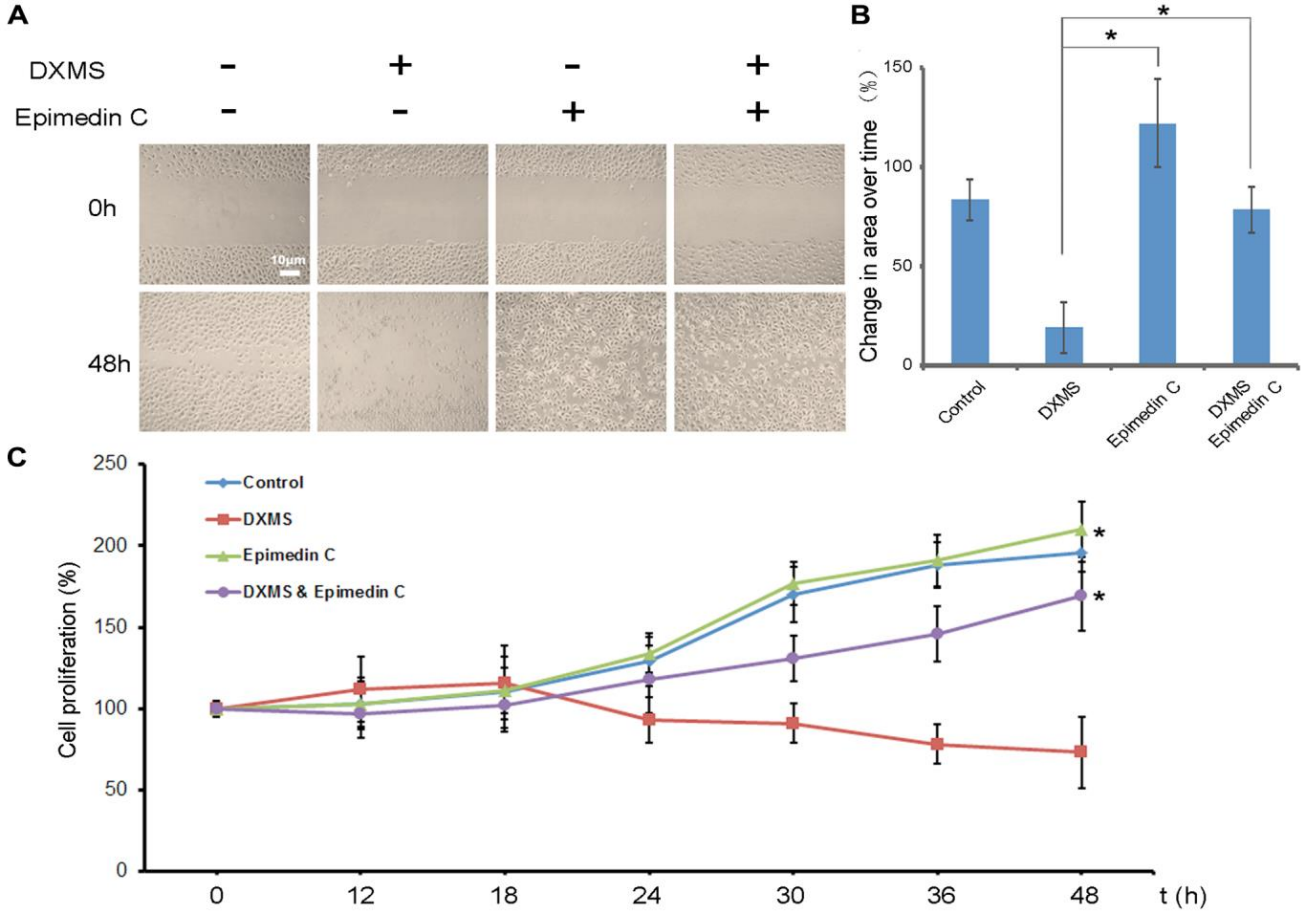
**Epimedin C enhances migration of osteoblasts in dexamethasone treatment.** (**A**) A Following scratch wound, application of Epimedin C accelerated migration of osteoblasts. (**B**) Change in area over time for osteoblasts at 48 h after treatment was quantified. *n* = 6 independent experiments. ^*^p < 0.01. (**C**) Cell proliferation was measured by BrdU assay. OB cells were seeded in a 96-well plate with serum at basal concentrations. Finally, 10 μM BrdU was added to the plate and cells were incubated for 4 hr. Data are shown as mean ± sd.

The function of Epimedin C in stabilizing OB growth following DXMS treatment was revealed at present study. DXMS-treated cells exhibited a slow growth pattern compared to non-treated cells, whereas the cells treated with Epimedin C exhibited growth at an average speed (Figure 1A). After DXMS treatment, compared with cells treated with DXMS alone, cells cultured with Epimedin C showed better growth and proliferation rate (Figure 1B, Column 4, Figure 1C) indicating that Epimedin C enhances the migration and growth after DXMS treatment.

### DXMS suppresses RhoA expression by inhibiting its transcription in OBs

**Figure 2:**
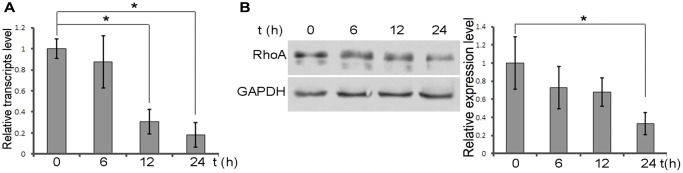
**DXMS suppress the expression of RhoA.** (**A**) Total RNAs were extracted from OB at 6, 12, 24 h post treatment DXMS. The relative transcripts level verified by qPCR. (**B**) Protein level of RhoA confirmed by immunoblot.

We then explored the potential molecular target in DXMS-induced OB. DXMS was added to the culture medium and the OBs were harvested to evaluate RhoA expression. The results revealed that DXMS treatment had a negative modulation effect on RhoA expression (Figure 2A). DXMS remarkably suppressed the mRNA level after treatment for 24 h (Figure 2A column 4). 6–24 h after DXMS treatment, the protein level also confirmed the low RhoA level, revealing the negative impact of DSMS on RhoA protein level (Figure 2B; columns 2, 3 and 4). The synchronous pattern of mRNA and protein levels is important evidence that DXMS inhibits RhoA expression at the beginning of transcription.

### DXMS suppresses RhoA expression though Nrf1 modulation in OBs

**Figure 3:**
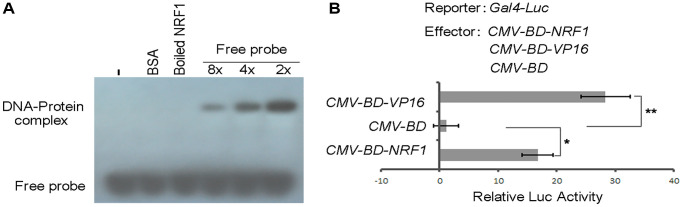
**DNA-binding and transcription activation activity of NRF1.** (**A**) EMSA analysis of the binding of NRF1 to RhoA promoter. Proteins were incubated with biotin-labeled probe in the absence or presence of 8- to 2-fold of unlabeled probes for 30 min. (**B**) DLR assay of NRF1 transcription factor activity in OB. Asterisks indicate statistically significant differences as determined by Student's *t*-test (^*^P < 0.05, ^**^P < 0.01).

To investigate upstream molecules that regulate the transcription of RhoA, Nrf1 was identified by the DNA pull-down assay and the coding region of Nrf1 was cloned and constructed in the pET28a (+) vector and then expressed in BL21 (DE3) for Nrf1 protein. Apparently, the protein exhibits a strengthen signal with a decreased concentration of the cold probe (Figure 3A).

**Figure 4:**
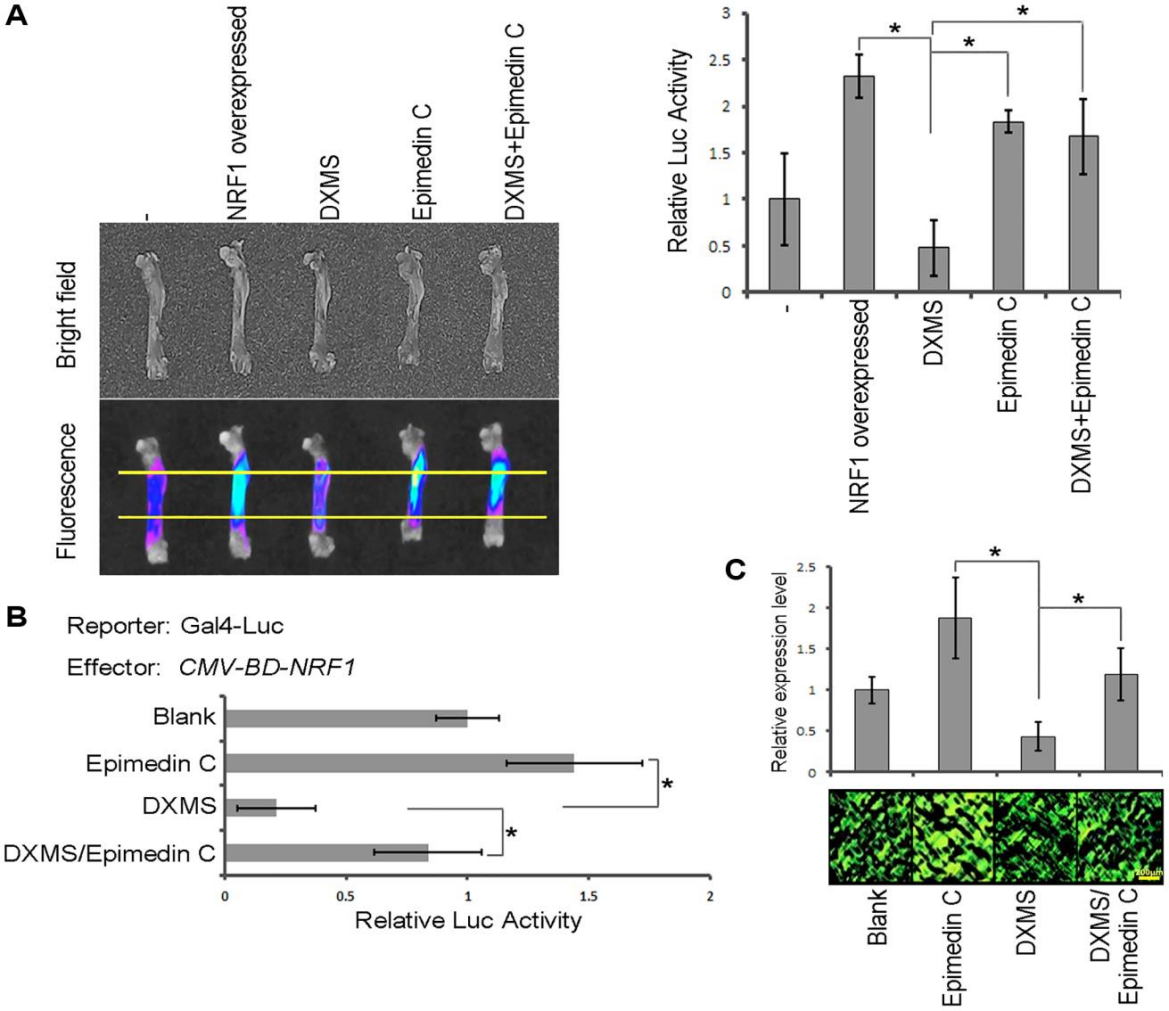
**Epimedin C antagonizes the effects of DXMS.** (**A**) Transient expression assay. Reporter Luc drive by RhoA promoter. (**B**) DLR assay with treatment of DXMS and/or Epimedin C. (**C**) IF staining for the endogenous RhoA in rodent long bone slice.

The transcription factor activity of Nrf1 was examined using a dual-luciferase reporter (DLR) assay system in cultured cells. The coding sequence of Nrf1 was fused to the DNA sequence encoding the GAL4 DNA-binding domain under the control of the cytomegalovirus (CMV) promoter in pcDNA3.1. As shown in Figure 3B, Nrf1 was able to activate the reporter expression *in vivo* compared with the negative control (*CMV-BD*). Results with the transient expression assay also showed that overexpression of Nrf1 in rodent long bone shaft presents a higher Luc activity (Figure 4A, column 2) in comparison with the non-transgenic ones, revealing that Nrf1 promotes the RhoA promoter drive-Luc signal.

### Epimedin C antagonizes the effects of DXMS induced OBs

Epimedin C was added to the culture medium externally and cell growth was determined in this study. A straight gap was made manually by scratching the surface of the cells, and the gap area was calculated. In the Epimedin C-treated groups, the migration rate was normalized and antagonized the influence of DXMS (Figure 1A and 1B).

A similar phenomenon was also visualized at the molecular level using DLR assays. The results revealed that Nrf1 activation was also enhanced by Epimedin C (Figure 4B, column 4) co-cultured with DXMS, which obviously functioned as an antagonist in the cellular model.

A transient expression assay was used to determine the Epimedin C function and the long bone shafts of rodents were sampled as the target region to calculate Luc reporter activity. The bones from mice administered Epimedin C were found to exhibit more intense Luc activity compared to those obtained from DXMS-treated mice (Figure 4A). Effect of Epimedin C on endogenous RhoA was evaluated by IF staining. The protein level of RhoA was remarkably decreased by external DXMS treatment, whereas Epimedin C restored the expression of RhoA (Figure 4C). Thus, our results confirmed that Epimedin C antagonized the effect of DXMS both *in vitro* and *in vivo*.

### RhoA expression level was down regulated in CRISPR *nrf1* cells

**Figure 5:**
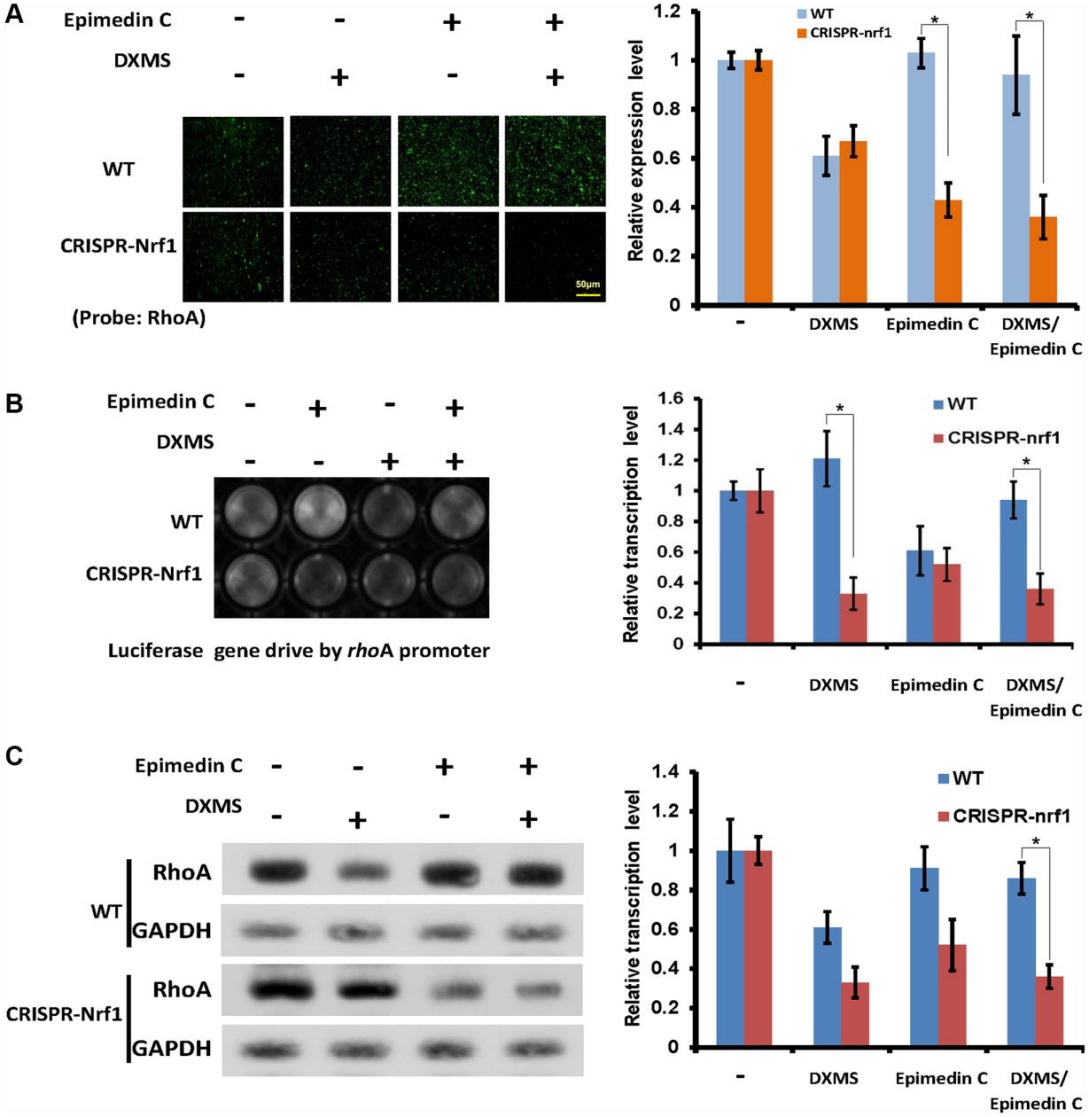
**Epimedin C lost its function without Nrf1 expression.** (**A**) OB cells probed by RhoA antibody, WT and CRISPR-Nrf1 treated with Epimedin C and DXMS in different dosage. (**B**) The Luc signal captured in OB cells. WT and CRISPRE-Nrf1 group treated with Epimedin C and DXMS, the Luc ORF drive by RhoA promoter exhibits the positive signal in different level. (**C**) Compared with WT group, the CRISPR-Nrf1 OB cells got a lower expression level of RhoA protein, even in the Epimedin C treatment condition.

Internal Nrf1 expression was verified in CRISPR-*nrf1* and the Osteoblast cells were also selected as samples to detect RhoA promoter activation. The RhoA reading frame was replaced by reporter Luc in RhoA promoter assay experiment. The origin signal RhoA in living Osteoblast was examined by fluorescence microscope, the protein RhoA was probed by antibody (Abcam, ab271951), as shown in Figure 5A. The RhoA signal in WT cells was as same as described in DXMS and Epimedin C treatment, while the signal in CRISPR group was lower than group DXMS/Epimedin C and Epimedin C treatment. These two groups can also be read but the Epimedin C exhibited lost its salvage function that the RhoA expression level was still lower in front of DXMS treatment in CRISPR-*nrf1* cells.

RhoA promoter activation was also assayed in these Osteoblast cells, the reporter Luc activity was read and shown that the CRISPR-*nrf1* Osteoblast was not responding to the Epimedin C, and did not salvage the RhoA promoter activation in DXMS treatment (Figure 5B).

The native RhoA expression level was also examined in WT and CRISPRE-*nrf1* OB cells, with and without the expression of Nrf1, the native RhoA level is different. The results can be verified as shown in Figure 5C, that with the comparison of WT cells, CRISPR-*nrf1* cells got lower density of RhoA signal, although treated with Epimedin C only.

## DISCUSSION

OP results in the weakening of bones and causes severe fractures, particularly at age over 50 years. In this study, we found that RhoA was increased in OBs and OP models, and revealed that Nrf1 is a transcription factor responding for modulating RhoA promoter, but most importantly the Epimedin C could protect the DXMS-induced OP.

Skeletal health relies on structural integrity and sufficient bone mass. The balance between bone formation and bone resorption is maintained through a tightly regulated equilibrium by OBs and osteoclasts (OC). OBs play a role in bone development via differentiation into osteocytes and prohibiting OC formation by producing enough osteoclastogenesis inhibitory factor (OPG) to inhibit OC growth [30–32]. Key endogenous factors or pathways in OBs contribute to the protection against OP. In the present study, we found that the RhoA was decreased in the DXMS-induced OP. That is probably a potential reason why DXMS could induce OP in other models [33, 34]. RhoA contributes significantly to OB stability, differentiation, proliferation, and survival [35].

The inhibition of RhoA expression indicated the disturbed balance between bone formation and bone resorption, thus the excessive activity of OC could lead to the damage of bone formation. RhoA is crucial in the survival of OBs in normal as well as in individuals with OP. The crucial role of RhoA has been reported in the RhoA/ROCK signaling pathway to regulate cytoskeletal dynamics and is essential for the proliferation, differentiation, and survival of OBs [36–39]. Treatment with DXMS caused the OBs to become disordered and grow abnormally, resulting in cell death, this phenomenon has been verified in our cell model, also reported in other articles [40, 41]. In our study, DXMS treated cells exhibit decreased expression of RhoA, that made the OP cell model establish *in vitro*. This model provides a cellular platform for verifying the drug properties of Epimedin C. Finally, the pathway that Epimedin C influenced in OB cells were found and confirmed, phenotype effects also have been verified. Our results have shown that, Epimedin C inhibited this toxicity and salvage the cells from DXMS, verifying the role of Epimedin C in the treatment of DXMS-induced OBs. To investigate the molecular mechanisms of Epimedin C in OBs, the promoter region of RhoA was cloned and labeled with biotin to screen the candidate upstream molecule. The RNA pulldown screening assay in this paper was performed to find out the binding protein that acted as transcription factor or co-factors in DNA-protein interaction level. A potential target and a putative transcription factor, Nrf1, was identified using MS and verified by EMSA and the DLR. The Nrf1 has been reported as an essential factor produced by OBs [26] and the participation of Nrf1 in the regulation of osterix expression, OB targeting, and bone formation, as a crucial regulator of normal function was also reported [25]. In the present study, we found that Nrf1 could directly bind to the promoter region of RhoA and regulate its transcription. This result provides an idea of the therapeutic targeting of Nrf1 and suggests a novel treatment for OP by balancing the Nrf1-DNA relationship. The binding site probably got a specific motif that is responsible for DNA-protein recognition and binding. While the screening and related data of motif is not shown in this paper, the motif will be better investigated in future research. Our findings further support the potential therapeutic approach of Epimedin C in DXMS-induced OBs. The finding that Nrf1 responded to Epimedin C but not affected by DXMS is of importance, because this response stabilizes mRNA levels by the recovery of RhoA transcription and ensures the maintenance of protein levels. Our finding suggests that once the antagonism exists between DXMS and Epimedin C in OB cells, Nrf1-RhoA could be considered a core node between the two external stimulators. After treatment with Epimedin C, when Luc transcription was determined, the signal from the middle long bone shaft was found to be stronger than that from the DXMS-treated cells. The signal from the rodent long bone shaft slices provides evidence for such transient expression. The specificity of Nrf1 in transcriptional regulation of RhoA probably also relies on post-translational modifications and its interaction with other co-factors. Although Epimedin C antagonizes DXMS induced OP in rodents, clinical trials are needed for further confirmation of its safety and efficacy.

Nrf1 was verified an essential element for Epimedin C impacting the RhoA expression process, the CRISPR approach revealed that the internal RhoA level was first detected in CRISPR cells compared with WT Osteoblast cells, showing that the RhoA level was downregulated and cannot salvage by Epimedin C with the DXMS treatment, suggesting that DXMS treatment will have a persistent effect on OB cells.

One speculation is that the RhoA promoter activation was fragile due to the lack of Nrf1. In our study, the reading frame of RhoA ORF was replaced by reporter gene Luc, with the original RhoA promoter. The result shows that the promoter activity is no longer as strong as it in normal condition, when the Nrf1 content was leveled down. At this status the Epimedin C lost its salvage ability in maintaining the RhoA expression. The native RhoA was not salvaged by Epimedin C in the DXMS treatment confirming that the Nrf1 is highly required in this process. The present study provides detailed confirmation that Epimedin C could function as a reagent antagonistic to the DXMS in OB cells and its possible mechanism is to enhance the binding of Nrf1 and RhoA promoter region that salvage the negative effect of DXMS.

In conclusion, our study confirms the positive function of Epimedin C in OBs that antagonizes the DXMS via stabilizing the Nrf1-RhoA promoter complex that protects OBs from the damage of DXMS induced cell death. The results from this study provide possibilities the Epimedin C could be an adjuvant chemical in osteoporosis therapy. The potential clinical values of Epimedin C were verified in rodent and OB cell model, it provides a theoretical clue and reliable reference for basic research and its application.

## MATERIALS AND METHODS

### Cell isolation and culture

Mus musculus OBs were isolated from the long bones of 6-week-old mice, according to the protocol described by Bakker and Klein [42]. Cells were washed with PBS (Gibco, 10010-023, China) and centrifuged at 300 × g for 5 min, then inoculated into 25 cm^2^ flasks and cultured at 37°C. Dulbecco’s modified eagle medium (DMEM, Gibco, 11885-092, China) supplemented with L-glutamine, and pyruvate was used for culture OBs.

An *in vitro* scratch assay was designed based on the method reported by Mouritzen [43]. OBs were isolated from rodents for 4 weeks, inoculated in flasks, and cultured with or without Epimedin C and DXMS. A manual scratch was made by the pipette tip in the middle of the culture flask when the cell was grown up.

### Animals

Wildtype C57BL/6N (Jackson Laboratory, Bar Harbor, USA) male mice aged 4 weeks with body weights ranging from 20–25 g were used for all experiments. The mice were housed in a temperature-controlled room (20–24°C) and subjected to a 12 h light/dark cycle and provided free access to standard rodent chow and drinking water.

### DNA delivery

JetPEI reagent (VWR, 89129-920, USA) was used to transiently express the *Luc* reporter gene driven by the Rho1 promoter in a rodent model. Primers used in promoter cloning were shown in Table 1. The modified *pcDNA3.1*-*RhoA pro*-*Luc* (50 μg) vector was mixed with *in vivo*-jetPEI reagent in 400 μL of 5% glucose solution and injected into the tail vein of mice for *in vivo* experiments. Bioluminescence imaging of luciferase expression in bone tissue was performed using the NightOWL II LB 983 imaging system (Berthold Technologies, Bad Wildbad, Germany).

**Table 1:** Primers used in this paper.

**Primer sequence**	**Names and purpose**
AGCCAGCGGACCCAGCGATATCTCTGGAGA	*Mus* RhoA promoter, cloning
CCGAATCACTCATCCTTTAAGCAAGCAGGG	*Mus* RhoA promoter, cloning
ATGGAGGAGCACGGAGTGACCCAAACTGAAC	*Mus* Nrf1 ORF, Cloning
CTGTTCCAAGGTCACCACCTCCACAGCCTGGCCA	*Mus* Nrf1 ORF, Cloning
CTGGGCCACGTTACAGGGCG	*Mus* Nrf, qPCR
AGCTGCCACTGCGGCTTCTG	*Mus* Nrf, qPCR
ACGAGCACACGAGACGGGAGT	*Mus* RhoA, qPCR
AGCAGCTCTCGTGGCCATCTCA	*Mus* RhoA, qPCR
ATGGCTGCCATCAGGAAGAAACTGGTGATTGTTGG	*Mus* RhoA ORF, Cloning
CAAGATGAGGCACCCAGACTTTTTCTTCCCACGTCT	*Mus* RhoA ORF, Cloning

### DXMS and Epimedin C treatment

DMEM medium was mixed with DXMS at a concentration of 10 μM, pre-warmed at 37°C, and loaded into the culture flask. Cells were sampled at 0, 1, 6, 12, and 24 h. Epimedin C was dissolved in H2O, and the final concentration was 10 μM. The liquid was orally administered by dropping it directly into the mouth at a specified dosage. DXMS was dissolved in drinking water at a concentration of 10 μM and administered at a final dose of 1 × 10^−4^ μmol/mouse/day. Oral administration was performed for 4 weeks. A similar procedure was used for the administration of Epimedin C at a final dose of 1 × 10^−3^ μmol/mouse/day.

### BrdU (Bromodeoxyuridine) cell proliferation assay

Detection of BrdU incorporation was performed by ELISA (Cat. No. #6813, Cell Signaling Technology, Danvers, MA, USA) to investigate the proliferation of OBs cell lines according to the manufacturer’s instructions. 5000 cells were seeded in 100 μl starvation medium (DMEM, 0.1% FBS, 100 U/ml penicillin and 100 mg/ml streptomycin) and incubated at 37°C for 24 h hours to synchronize the cell cycle. Medium was then changed to 1x BrdU solution prepared in regular growth medium (DMEM, 5% FBS, 100 U/ml penicillin and 100 mg/ml streptomycin) cultured for 6 h at 37°C to induce proliferation. 10 μM DXMS and 10 μM Epimedin C were used in cell treatment for 0–48 h. Subsequent procedure was performed according to the manufacturer’s instructions. The BrdU incorporation was measured at 450 nm with the TECAN GENios V4.62-07/01 microplate reader (Tecan, Reading, UK) and XFLUOR4 Version V 4.51 software (Tecan).

### Real time-PCR

Total RNA was extracted using the PureLink^™^ Total RNA Kit (Invitrogen, IS10006) according to the manufacturer’s instructions, and cDNA was synthesized using SuperScript^™^ III First-Strand Synthesis SuperMix (Invitrogen, 11752050). The marker gene primers synthesized by Integrated DNA Technologies, Inc. (IDT) and the sequences are shown in Table 1. The results were quantified using the 2^−ΔΔC^ method by Ish-Shalom and Lichter [44]. Primers used in qPCR were shown in Table 1.

### Immunoblotting

Whole protein lysates were separated using SDS-PAGE and transferred to PVDF membranes. The blots were incubated with antibodies (Cell Signaling Technology, Inc) directed against RhoA (26C4), GAPDH (D16H11), NRF1 (D9K6P), followed by incubation with the corresponding HRP-conjugated secondary antibody and detected using the Pierce^™^ Fast Western Blot Kit (35050). The band intensities were quantified using ImageJ software (National Institutes of Health, USA).

### DNA pull-down

The promoter region was amplified according to the NCBI Reference Sequence: NG_051308.1 and a 2,100 bp target DNA was acquired with a 5′ biotin label. The procedure for DNA pull-down screening was optimized using the procedure reported by Iwasaki [45] and Larsen [46]. The mixture of the DNA probe and total protein extracted from rodent OBs were incubated overnight at 4°C. The DNA-protein complexes were washed with Binding/Wash Buffer (Tris-buffered saline containing 0.1% Tween-20 detergent) and harvested using Pierce^™^ streptavidin magnetic beads (88816).

The RhoA promoter was cloned and labeled with biotin adhered to streptavidin magnetic beads used as bait. Total protein extracted from cultured OBs was mixed with the bait and incubated. Candidate proteins in the elution of the DNA pull-down were identified using MS. Nrf1 was identified among the candidate binding proteins. Next, the binding ability was confirmed using EMSA.

### MS sample processing and analytical methods

The samples prepared in this study were modified based on the method reported by Hou [47]. The sample was precipitated by adding five volumes of cold acetone and incubating overnight at −20°C. The precipitated protein was washed twice with 80% cold acetone. Then, 10 μL each of 6 M urea, 2 M thiourea, and 100 mM ammonium bicarbonate were added to the pellet. The solubilized protein was reduced by DTT and alkylated by IAA. Next, trypsin was added for digestion and allowed to react overnight. The digested peptide was C18 zip-tip desalted, lyophilized, and suspended in 10 μL of 5/0.1% acetonitrile/formic acid.

### LC-MS+MSMS (TimsTOF pro)

Suspended peptide (1 microliter) was loaded onto a C18 column and eluted using a stepwise gradient with acetonitrile at a flow rate 400 nL/min. A Bruker nanoElute system linked to a time TOF Pro mass spectrometer was used. The LC gradient conditions were as follows: the initial conditions were 2% B (A: 0.1% formic acid in water, B: 99.9% acetonitrile, 0.1% formic acid), followed by a 4.5 min ramp to 17% B. 17–25% B over 8.5 min, 25–37% B over 4.5 min, a gradient of 37–80% B over 2 min, hold at 80% B for 5.5 min. The total run time was 25 min. MS data were collected over an m/z range of 100 to 1700. During MS/MS data collection, each TIMS cycle included 1 MS + an average of 10 PASEF MS/MS scans. The raw data were searched using PEAKS with the Uniport Mus/Homo protein database. Data were searched with trypsin as an enzyme; two missed cleavages were allowed, carbamidomethyl cysteine was a fixed modification, oxidized methionine, acetylation on protein N terminus variable mod, 15 ppm mass tolerance on precursor ions, and 0.1 Da on-fragment ions.

### Electrophoretic mobility shift assay (EMSA)

EMSA was carried out using biotin-labeled probes and the Pierce Light Shift Chemiluminescent EMSA kit (Thermo Scientific, 89880, Rockford, IL, USA). His-tagged Nrf1 proteins were expressed and purified. Promoter sequences and synthesized DNA fragments were used as probes. Unlabeled DNA fragments were used as cold probes. The binding reaction was carried out in a 20 μl reaction mixture at room temperature for 30 min and then separated by 6% native polyacrylamide gel in 0.53 Tris-borate/EDTA buffer. The bands were detected according to the instructions provided with the EMSA kit.

### Dual-luciferase reporter (DLR) assay

To verify transcription factor activity in OBs, the coding region of the *nfe2l1* gene was cloned into the expression vector pcDNA3.1-BD to generate the BD-*nfe2l1* effector plasmid. Gal4-Luc was used as a reporter in the same backbone. The *Renilla* Luc gene served as an internal control. The DLR assay was conducted according to the method described by Ohta [48]. OBs were transfected using a Lipofectamine 3000 (Thermofisher, L3000015, Carlsbad, CA, USA) -mediated transient expression system. The co-transformed cultures were kept in the dark for 16 h at 37°C. Luciferase assay was performed using the Promega DLR assay system and values were measured using a GloMax 20–20 luminometer (Bio-Rad, Hercules, CA, USA).

### CRISPR vector construction and cell verification

Original vector containing Cas91.1 expression frame and gRNA editing region purchased from Addgene (#71814), gRNA designed online and cloned into vector Cas91.1, verified by sequencing. The final sequence of Nrf1 (XM_027775873.1) gRNA is 5′-GGATTAGACTCAAACACGTG-3′ according to its score calculated on https://www.genscript.com/gRNA-design-tool. Purified plasmid of the final version was transfected into cells by JetPEI reagent as described in above chapter.

The positive signal, e.g., EGFP was verified by fluorescence microscope. Also, the signal in living cells e.g., Luc activity can be examined in the 96-well culture plate by adding additional Luciferin substrate, and the signal was read by NightOWL II LB 983 system (Berthold Technologies).

### Promoter isolation and transient expression assay

An *in vivo* experiment was also designed to verify the Nrf1 function in the RhoA promoter. A transient expression assay was performed using the luciferase coding region as a reporter. Luc fluorescence signals were captured using a low-light cooled CCD camera. The promoter sequence of the RhoA gene was cloned by PCR-based genome walking. The infusion system was used following the instruction manual (Clontech, San Jose, CA, USA) for the construction of vectors. The 1,193 bp promoter sequence was cloned into the pcDNA3.1 vector containing a Luc reporter gene to generate *RhoA Pro*: *Luc* as reporters. The coding sequence of *nfe2l1* gene was cloned into normal pcDNA3.1 to generate the constructs as effectors. For the expression assay in bone tissue, a JetPEI-mediated transfection system was used for rodent transfection and the bone tissue was isolated as samples to verify the expression of the *Luc* reporter. The detached bone shafts were sprayed with 1 mM D-luciferin (Promega, P1042), the Luc signal was captured using a low-light cooled CCD camera and the relative Luc activity was measured. Quantitative analysis was performed using IndiGo software (Berthold Technologies, Oak Ridge, TN, USA).

### Rodent long bone shaft immunofluorescence staining

Rodent long bone shafts were dissected and sliced according to the process described by Amend [49]. The middle of the tissue with the highest density was sliced as samples for observation. The primary antibody (Cell Signaling Technology, 2117) and the secondary antibody conjugated with Alexa Fluor 488 conjugated (Cell Signaling Technology, 4412) for IF staining observation.

The figures shown in this study, error bars represent the SD of three biological replicates with three technical repeats each. Asterisks indicate statistically significant differences as determined by Student’s *t*-test (^*^P < 0.05, ^**^P < 0.01).
